# A digital self-care intervention for psychological distress associated with premenstrual syndrome: a fully online controlled trial using alternating allocation

**DOI:** 10.1186/s12905-026-04539-3

**Published:** 2026-05-20

**Authors:** Yumie Ikeda, Miho Egawa, Takuma Ohsuga, Eiji Nakatani, Kaori Tsuyuki, Yoshimitsu Takahashi, Takeo Nakayama, Masaki Mandai

**Affiliations:** 1 Department of Health Informatics, Kyoto University School of Public Health, Konoe-cho Yoshida, Sakyo-ku, Kyoto, 606-8303 Japan; 2 Umisora Clinic Kyoto, Medical Corporation Kokoshikakai, Kyoto, Japan; 3 Department of Gynecology and Obstetrics, Kyoto University Graduate School of Medicine, Kyoto, Japan; 4 Department of Biostatistics and Health Data Science, Graduate School of Medical Science, Nagoya City University, Nagoya, Japan; 5 Department of Implementation Science in Public Health, Kyoto University School of Public Health, Kyoto, Japan

**Keywords:** Mobile Applications, Premenstrual Syndrome, Self Care, Stress, Psychological, Telemedicine, Women’s Health

## Abstract

**Background:**

Premenstrual syndrome (PMS) is associated with significant psychological distress and productivity loss in women. This study aimed to evaluate whether a low-intensity digital intervention—comprising a symptom-tracking smartphone application and standardized informational emails—could reduce the psychological burden of PMS among women who self-identified as experiencing PMS.

**Methods:**

We conducted a fully internet-based, open-label, parallel-group, nonrandomized controlled trial using centrally administered alternating allocation among women aged 18 years or older in Japan who self-identified as experiencing PMS. Participants were allocated alternately to either a 3-month intervention group (daily symptom tracking using a smartphone app and twice-weekly informational emails) or a waitlist control group. The primary outcome was the between-group difference in change scores on the psychological subscale of the Japanese version of the PMS-Impact Scale, measured from baseline to 3 months. Analyses were conducted per protocol.

**Results:**

A total of 419 women were enrolled and assigned to either the intervention group (n = 210) or the waitlist control group (*n* = 209). Of these, 355 participants were included in the per-protocol analysis. The intervention group showed greater improvement in self-reported psychological distress than the waitlist control group. The between-group difference in change score, defined as intervention minus control, was − 1.37 points (95% CI, − 2.47 to − 0.27; *p* = 0.015).

**Conclusions:**

A simple, scalable digital intervention was associated with greater improvement in self-reported psychological distress among women who self-identified as experiencing PMS. These findings suggest that accessible, non–clinician-led tools may have potential as a self-management strategy for PMS, although causal interpretation is limited by the nonrandomized design.

**Trial registration:**

This trial was registered with the University Hospital Medical Information Network Clinical Trials Registry (UMIN-CTR) on September 26, 2022. Trial registration number: UMIN000048422.

**Supplementary Information:**

The online version contains supplementary material available at 10.1186/s12905-026-04539-3.

## Background

Premenstrual syndrome (PMS) comprises recurrent physical and psychological symptoms linked to the menstrual cycle and can markedly impair quality of life and work productivity. In Japan, surveys indicate that 40%–80% of menstruating women experience PMS‑related symptoms, and about 5% meet the criteria for premenstrual dysphoric disorder (PMDD) [1].

PMS symptoms, including depressed mood, anxiety, irritability, and physical discomfort, can interfere with daily and occupational functioning. In Japan, menstrual-related symptoms are estimated to cause an annual productivity loss of approximately 680 billion yen, primarily due to presenteeism—working despite reduced performance [2, 3]. Addressing PMS through accessible and effective interventions is therefore a priority.

Pharmacological and cognitive behavioral therapies (CBTs) are effective, but drug regimens often entail side‑effects and poor adherence. Internet‑based CBT (iCBT) has demonstrated benefits for PMDD [4]; however, its reliance on structured, clinician‑led interactions limits scalability and feasibility, particularly for women with milder symptoms.

Low‑intensity digital interventions—such as standardized informational e‑mails and app‑based symptom tracking—may offer a scalable alternative. Nevertheless, randomised trials evaluating such interventions remain scarce. Given the broad impact of PMS—particularly among working women,validating self‑directed, scalable strategies is important for public health.

## Materials and methods

### Study design and setting

We conducted a fully internet-based, open-label, parallel-group, nonrandomized controlled trial in Japan using centrally administered alternating allocation to evaluate whether a low-intensity digital intervention could reduce PMS-related psychological distress in this population. Eligible participants were Japanese women aged 18 years or older who acknowledged experiencing PMS symptoms. Participants were alternately allocated in a 1: 1 ratio to the intervention arm or the waitlist control arm and were followed for 3 months.

The intervention consisted of daily symptom tracking using a bespoke smartphone application and standardized lifestyle-related informational emails delivered twice weekly. All trial procedures, including recruitment, eligibility screening, e‑consent, allocation, intervention delivery, and outcome assessment were conducted entirely online without any in‑person contact or site visits. Owing to the nature of the intervention, blinding of participants or investigators was not feasible.

### Participants

Inclusion criteria were: (1) self‑reported physical or psychological PMS symptoms; (2) ability to read Japanese; (3) routine smartphone use enabling internet-based surveys and app tracking; and (4) provision of electronic informed consent via the study website. We excluded women who had modified pharmacological treatment for gynecologic or psychiatric conditions within the previous 3 months because clinical stability might not yet have been achieved. Women receiving stable ongoing treatment were eligible. The use of over-the-counter pain relievers or supplements was not restricted.

We recruited via the study website, social media (Twitter, Facebook), and printed flyers distributed at universities and medical facilities. Prospective participants completed an internet-based registration form, reviewed study materials (including an explanatory video), and then provided e‑consent. Enrolment was defined as provision of informed consent, fulfilment of eligibility criteria, and completion of the baseline questionnaire. Recruitment was stopped once this number was reached. Patients and the public were not involved in the design, conduct, or reporting of this trial.

### Intervention and comparator

Participants were allocated to either an intervention group or a waitlist control group. Those in the intervention group received a 3-month digital intervention consisting of two components: a smartphone application for daily symptom tracking and a series of standardized informational emails.

The bespoke app enabled daily tracking of PMS-related symptoms using the Japanese version of the Daily Record of Severity of Problems (DRSP) [5, 6] as well as menstrual flow. Additional features included a calendar for recording and predicting menstruation onset, graphical visualization of past symptom trends, and a free-text memo function. The app was designed to facilitate self-monitoring and awareness of symptom patterns.

In addition, participants in the intervention group received a total of 26 informational emails, delivered twice weekly over the 3-month period. Email content was first developed through a consensus meeting involving three gynecologists, three psychosomatic medicine physicians, and one clinical psychologist. A subsequent literature review confirmed consistency with current evidence. Of the 26 emails, 9 addressed nutrients and eating behaviors, 8 focused on interpersonal and psychological issues, 4 addressed physical activity, 4 addressed sleep and daily routines, and 1 provided an explanation of PMS pathophysiology. The emails were unidirectional, standardized, and sent simultaneously to all participants in the intervention group, with no individualized support provided by healthcare professionals. Email engagement was assessed by tracking whether participants clicked a confirmation URL embedded at the end of each message.

Participants in the waitlist control group received no intervention during the initial 3-month period but were offered the same app-based tracking and email series after the follow-up assessment was completed. Thus, all participants ultimately received the same intervention, with the timing being the only difference between groups.

Given the non-clinical, low-risk nature of the intervention, no predefined criteria for adverse events were established.

### Outcomes

The primary outcome was the between-group difference in change scores on the psychological subscale of the Japanese version of the PMS-Impact Scale, measured from baseline to 3 months. The original scale, developed in German, consists of 18 items representing two factors: psychological impact (9 items) and social impact (9 items) [7]. The Japanese version, adapted for use among Japanese women who self-identified as experiencing PMS, was developed in 2018 and validated through factor analysis [8]. The Japanese version comprises 13 items (9 psychological and 4 social), with the psychological subscale maintaining identical items to the original version. Each item is rated on a 5-point Likert scale ranging from 1 (not at all applicable) to 5 (very much applicable). Subgroup analyses were performed according to PMS severity, classified by the Premenstrual Symptoms Screening Tool (PSST) into none–mild, moderate–severe, and probable PMDD. [9] Within each stratum, mean changes in psychological subscale scores were compared between the intervention and control groups.

Secondary outcomes included between-group differences in change scores from baseline to 3 months on the following measures:

- Total score and social subscale score of the PMS-Impact Scale (Japanese version) [8]
- The Japanese version of the Valuing Questionnaire (VQ) [10]
- The Japanese version of the Self-Compassion Scale–Short Form [11]

All questionnaires used in this trial were previously published instruments. Validated Japanese versions were employed for all outcome measures, including the PMS-Impact Scale [8], the Daily Record of Severity of Problems (DRSP) [5, 6], the Valuing Questionnaire [10], and the Self-Compassion Scale–Short Form [11]. PMS severity classification was based on the Premenstrual Symptoms Screening Tool (PSST) [9], using a licensed Japanese translation provided by McMaster University (License No. PSST23-015), which has undergone translation and back-translation procedures but has not yet been formally validated in Japanese populations. No new questionnaire items were developed specifically for this study. Assessments were conducted via self-report questionnaires at two time points: baseline and 3 months post-enrollment.

### Data collection and follow-up

All data were collected online. Aside from the scheduled delivery of informational emails, no additional contact or support was provided during the 3-month intervention period. Outcome measures were assessed at two time points: baseline (before intervention) and at 3 months post-enrollment, using a secure internet-based survey platform.

Participants were given two weeks to complete the post-intervention assessments. During this period, up to two reminder emails were sent at 3-day intervals to nonrespondents. Although daily symptom data were collected through the app using the Japanese version of the DRSP, these data were not included in the present analysis.

All questionnaire responses were collected via Google Forms and anonymized by a third-party contractor before being transmitted to the research team. Data were stored securely and managed by the Department of Health Informatics, Kyoto University School of Public Health.

### Statistical analysis

Sample-size estimation assumed a small-to-medium effect (Cohen d = 0.30) for the primary outcome, the psychological subscale of the PMS-Impact Scale. Using a two-sided significance level of 0.05 and a statistical power of 80%, a total of 352 participants (176 per group) were needed to detect a between-group difference in change scores. To account for potential attrition, the target enrollment was set at 400 participants.

Between-group differences in change scores for the primary and secondary outcomes were analyzed using two-sample t-tests. For each outcome, change scores were calculated as the difference between baseline and 3-month values. The mean change in each group, the mean difference between groups, and two-sided 95% confidence intervals (CIs) were reported. Analyses were performed on the per-protocol set (PPS). Participants in the intervention group were included in the PPS if they met all of the following criteria: (1) used the symptom-tracking app on at least 27 of 90 days (≥ 30%), (2) opened at least 13 of 26 informational emails (≥ 50%), (3) completed the PMS-Impact psychological subscale at both baseline and 3-month follow-up. Participants in the waitlist control group were included if they completed the same outcome measures at both time points. Missing data were not imputed; only complete cases were analyzed. In addition to the primary analysis, subgroup analyses were performed based on baseline characteristics. The subgroups included PMS classification (None-to-Mild, Moderate-to-Severe, PMDD), use of psychotropic medication (Yes or No), employment status (Full-time vs. Not working or part-time), and marital status (Married vs. Unmarried). For each subgroup, the between-group difference in symptom score change (intervention minus control) and its 95% confidence interval were calculated to evaluate potential effect modification.

Furthermore, in addition to the primary per-protocol analysis, two supplementary analyses were conducted to assess the robustness of the findings. First, a full analysis set (FAS) analysis included all participants with available 3-month outcome data, regardless of adherence status in the intervention group. Second, a conservative sensitivity analysis approximating the intention-to-treat principle included all allocated participants except those who withdrew consent, with missing 3-month outcomes imputed under the conservative assumption of no change from baseline, that is, baseline values were carried forward. These analyses were performed to evaluate the potential impact of missing outcome data and adherence-based exclusions on the study results.

All analyses were conducted using Stata/SE version 15.1 (Stata Corp, College Station, TX, USA). Two‑sided p values < 0.05 were deemed statistically significant.

### Ethical approval and informed consent

The study was approved by the Ethics Committee of Kyoto University Graduate School of Medicine (approval No. C1566). All participants provided electronic informed consent before enrolment. All procedures complied with institutional and national ethical standards and with the 1964 Helsinki Declaration and its later amendments.

## Results

### Participants and baseline characteristics

**Fig. 1:**
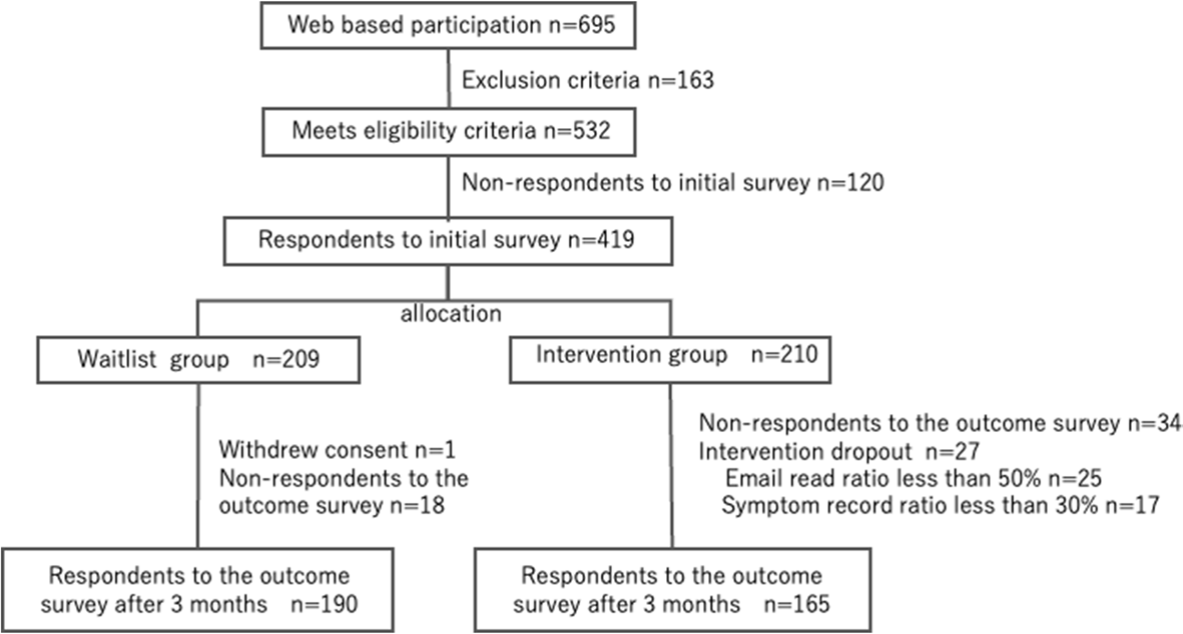
CONSORT Flow diagram of participant enrollment, allocation, follow-up. *Categories were not mutually exclusive

Participant enrolment began on 11 October 2022 and closed on 17 November 2022. A total of 419 participants completed internet-based registration and were alternately allocated to the intervention arm (*n* = 210) or the waitlist control arm (n = 209). Among those in the intervention group, 165 participants (78.6%) met per-protocol criteria and were included in the primary analysis. In the waitlist control arm, 190 participants (90.9%) completed outcome assessments at both time points and were included in the analysis (Fig. 1). Baseline characteristics are summarized in Table 1; no substantial between‑arm differences were observed for age, marital status, educational attainment, employment status, PMS severity, or baseline psychological‑impact scores.

**Table 1:** Participants background (Per-Protocol set)

		Intervention groupN=165	Control groupN=190
Age, mean（SD）		31.1 (8.1)	30.9(8.7)
Medication	Use of psychotropic medication, n (%)	13 (7.9)	9 (4.7)
	Use of oral contraceptives, n (%)	18 (10.9)	19 (10.0)
Operating System	iPhone, n (%)	126 (76.4)	141 (74.2)
	Android, n (%)	39 (23.6)	49 (25.8)
Marital Status Married, n (%)		78 (47.3)	86 (45.3)
Employment Status	Full time, n (%)	92 (55.8)	104 (54.7)
Education	Student (High school, vocational school, university, graduate school) , n (%)	42 (25.5)	53 (27.9)
	Middle school graduate, n (%)	0 (0)	1 (0.5)
	High school graduate, n (%)	13 (7.9)	17 (8.9)
	Vocational School/University Graduate, n (%)	87 (52.7)	97 (51.1)
	Post-graduate degree, n (%)	23 (13.9)	22 (11.6)
PMS classification (PSST)	None - Mild PMS, n (%)	32 (19.4)	35 (18.4)
	Moderate PMS, n (%)	75 (45.5)	98 (51.6)
	PMDD, n (%)	58 (35.1)	57 (30.0)

*Data represent no. (%) of patients unless otherwise specified

### Adherence and delivery fidelity

In the study (per‑protocol set), mean adherence to daily symptom tracking over 3 months was 96.3% (SD 11.5), and mean email open rate was 91.2% (SD 9.5). All digital components were delivered automatically as intended, with no protocol deviations.

Participants in both arms received reminder emails 14 and 11 days before the outcome‑survey deadline to encourage completion of the 3‑month follow‑up. No harms, complaints, or concerns were reported by participants.

### Primary outcome

**Fig. 2:**
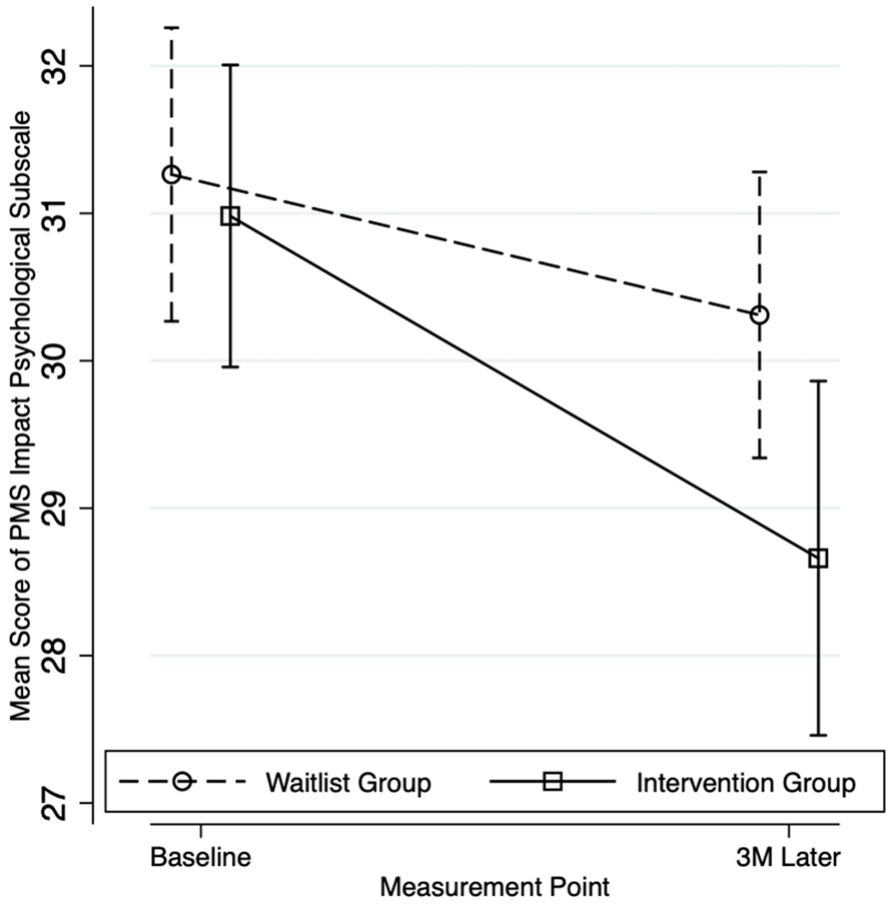
Group differences in change score of psychological subscale of premenstrual syndrome impact scale. *Error bars represent 95% confidence intervals for each point estimate

The primary outcome was the group difference in change in psychological burden associated with PMS, as measured by the psychological subscale of the Japanese version of the PMS-Impact Scale. From baseline to 3 months, the intervention arm (n = 165) showed a mean reduction of 2.32 points, whereas the waitlist control arm (n = 190) showed a reduction of 0.95 points. The between‑arm mean difference in change score, defined as intervention minus control, was − 1.37 points (95% CI, − 2.47 to − 0.27; p = 0.015), indicating greater improvement in the intervention group. Across all PMS severity strata, participants in the intervention group exhibited greater reductions in psychological burden at 3 months than those in the waitlist group (Fig. 2).

In the supplementary full analysis set (FAS) analysis, which included all participants with available 3-month outcome data regardless of adherence status in the intervention group, the intervention group (*n* = 176) showed a mean reduction of 2.00 points, whereas the waitlist control group (*n* = 190) showed a reduction of 0.95 points. The between-group mean difference in change score, defined as intervention minus control, was − 1.09 points (95% CI, − 2.17 to − 0.01), indicating greater improvement in the intervention group. In the conservative sensitivity analysis, which included all allocated participants except those who withdrew consent and imputed missing 3-month outcomes as no change from baseline, the intervention group (*n* = 210) showed a mean reduction of 1.70 points, whereas the control group (*n* = 208) showed a reduction of 0.87 points. The between-group mean difference in change score was − 0.84 points (95% CI, − 1.80 to 0.10). The direction of effect was consistent across analyses, although the estimated effect was attenuated and the confidence interval included the null value in the conservative sensitivity analysis. Baseline characteristics of participants included in and excluded from the primary per-protocol analysis are shown in Supplementary Table 1.

**Fig. 3:**
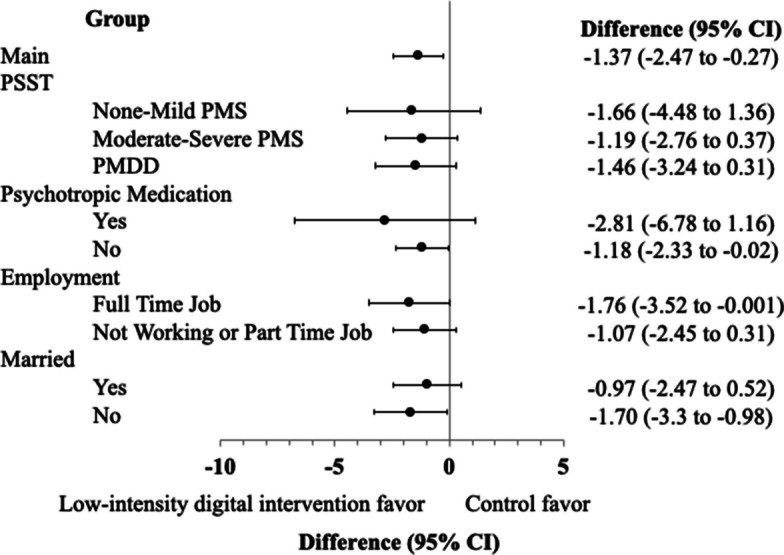
Group differences in change score of psychological subscale of premenstrual syndrome impact scale across subgroups. *This forest plot presents the mean differences (intervention − control) and 95% confidence intervals for score changes of psychological subscale of Premenstrual Impact scale classified by premenstrual symptom severity classification by Premenstrual Screening Tool, psychotropic medication use, employment status, and marital status. Between-group differences are shown as the change score in the intervention group minus the change score in the control group. Change score was calculated as the 3-month value minus the baseline value. For PMS-Impact Scale outcomes, negative values indicate greater reduction, and therefore greater improvement, in the intervention group. *PSST: Premenstrual Screening Tool; PMS: Premenstrual syndrome; PMDD: Premenstrual Dysphoric Disorder

Subgroup analyses revealed a consistent trend toward greater improvement in PMS symptom scores in the low-intensity digital intervention group compared to the control group across most subgroups (Fig. 3). Greater improvement was observed, particularly among those not taking psychotropic medications, full-time employees, and married participants.

### Secondary outcomes

Secondary outcomes included changes in total and social subscale scores on the PMS-Impact Scale, as well as scores on the Valuing Questionnaire (VQ) and the Self-Compassion Scale–Short Form.

- PMS-Impact Total Score: The intervention arm improved by 3.10 points versus 1.35 points in the control arm, yielding a between-arm difference in change score of −1.75 points (95% CI, −3.19 to −0.32), indicating greater improvement in the intervention group.
- PMS-Impact Social Subscale: Although the intervention arm improved by 0.78 points compared with 0.40 points in the control arm, the between-arm difference in change score was −0.38 points (95% CI, −1.00 to 0.24), which was not statistically significant.
- Valuing Questionnaire (VQ): The Valuing Questionnaire score increased by 1.04 points in the intervention arm and 1.19 points in the control arm; the reported between-arm difference in change score was −0.15 points (95% CI, −1.47 to 1.16).
- Self-Compassion Scale–Short Form: The Self-Compassion Scale–Short Form score increased by 0.52 points in the intervention group and 1.15 points in the waitlist control group; the between-group difference in change score was −0.64 points (95% CI, −1.62 to 0.35), suggesting that changes in self-compassion were not greater in the intervention group.

## Discussion

In this nonrandomized controlled trial with alternating allocation, participants who received a low-intensity digital intervention, comprising a symptom-tracking smartphone app and standardized informational emails, showed greater improvement in self-reported psychological distress associated with PMS than those in the waitlist control group. The intervention was simple, non-clinician-led, and did not include personalised feedback, yet it was associated with greater improvement.

The most notable finding was the pronounced improvement in the psychological sub-scale of the PMS-Impact Scale among intervention participants, despite significant gains in the total score. This finding suggests that, even without therapist involvement, the observed between-group difference is consistent with the possibility that digital tools promoting self-monitoring and general lifestyle guidance may contribute to improvements in PMS-related psychological burden. These findings are broadly consistent with previous studies suggesting potential benefits of digital and internet-based interventions for premenstrual and mood-related conditions, including internet-based cognitive-behavioral therapy (iCBT) for PMDD and smartphone-based mental health interventions [4, 12]. Unlike many previous digital interventions, however, the present intervention was fully automated, non–clinician-led, and low intensity, consisting only of app-based symptom tracking and standardized informational emails.

High adherence may have contributed to the observed benefits: 96% of participants in the intervention arm completed daily symptom tracking. Two mechanisms may underlie the observed effect. First, self-monitoring may exert therapeutic effects by fostering emotional awareness and cognitive distancing from distressing symptoms [13, 14]. This may partly explain the consistently high placebo-response rates observed in PMS trials in which symptom diaries are used in both study arms [15–17]. Second, the twice-weekly emails may have encouraged continued symptom tracking while also alleviating psychological stress by providing actionable advice. Together, these components may have amplified each other’s effect. Symptom self-monitoring may increase awareness of cyclical symptom patterns and help participants cognitively distance themselves from distressing experiences, while standardized psychoeducational emails may provide practical coping strategies and encourage continued engagement with self-monitoring.

In contrast, the intervention had no significant effect on social functioning or secondary psychological outcomes. This discrepancy may reflect the greater complexity and context-dependence of social and behavioral domains, which may require longer intervention durations or individualized support to show measurable change.

Because the primary per-protocol analysis excluded participants based on adherence in the intervention group, it may have selected more engaged participants and overestimated the intervention effect. The supplementary analyses showed a similar direction of effect, but with attenuated estimates. In the conservative sensitivity analysis, the confidence interval included the null value. Therefore, the findings should be interpreted cautiously.

These findings have practical implications. Unlike many previous digital interventions, our intervention was fully automated, non–clinician-led, and required minimal user burden. This low-intensity structure may enhance scalability in real-world settings. These findings are consistent with the growing interest in scalable digital self-care approaches to address common mental and behavioral health concerns at the population level [18].

However, because the present study used a nonrandomized design and assessed perceived psychological impact rather than symptom severity itself, direct comparison with randomized trials should be made cautiously.

A novel contribution of this study is that the findings are consistent with a possible psychological benefit of symptom tracking, although this mechanism could not be isolated in the present design. If confirmed, this mechanism could be leveraged in future intervention designs and improve understanding of placebo effects in PMS trials.

### Limitations

This study has several limitations. First, although the study was originally planned to use computer-generated random allocation, participants were ultimately assigned using an alternating sequence because of technical issues. Alternating allocation is not equivalent to randomization because the upcoming assignment can be predicted. Therefore, allocation concealment was limited, and the possibility of selection bias cannot be ruled out. In particular, if the upcoming assignment was known or could be inferred during enrollment, the enrollment or allocation process may have been influenced, even unintentionally. Although all procedures were conducted fully online and baseline characteristics appeared broadly balanced between groups, these features do not eliminate the possibility of unmeasured confounding or residual imbalance. Accordingly, the observed between-group differences should be interpreted cautiously as associations rather than definitive causal effects.

Second, the study design did not isolate the independent effects of the app and email components. Future studies should consider factorial designs. Third, outcomes were assessed via self-report questionnaires, which may limit generalizability. Fourth, participants were not clinically diagnosed with PMS using prospective symptom tracking over two menstrual cycles, which is considered the clinical standard. Instead, eligibility was based on self-identification of PMS symptoms; therefore, diagnostic misclassification cannot be excluded. In addition, although validated Japanese versions were used for most outcome measures, the Japanese translation of the Premenstrual Symptoms Screening Tool (PSST), which was used for PMS severity classification, has not yet undergone formal psychometric validation in Japanese populations. Furthermore, the primary outcome assessed the perceived psychological impact of PMS rather than symptom severity itself. Thus, the observed improvements may reflect changes in participants’ perception, coping, or appraisal of symptoms rather than direct reductions in the underlying symptoms. These measurement limitations should be considered when interpreting the findings. Lastly, the intervention period was limited to three months; the durability of benefits remains uncertain and warrants longer-term follow-up. Despite these limitations, this study provides preliminary evidence that a low-cost digital self-care intervention is associated with greater improvement in PMS-related psychological burden.Taken together, the findings highlight the potential of ultra‑low‑cost digital self‑care tools as a first‑line option for women with subclinical or mild PMS, and as an adjunct to clinician‑directed therapy in primary‑care settings.

## Conclusions

In this alternately allocated nonrandomized controlled trial, a simple, low-intensity digital intervention combining a symptom-tracking app and standardized informational emails was associated with greater improvement in self-reported psychological distress among women who self-identified as experiencing PMS. However, these findings should be interpreted with caution because of the nonrandomized design, the primary per-protocol analysis, and the use of self-reported perceived psychological impact rather than direct measures of underlying symptom severity. Although no significant improvement was observed in social functioning, the findings suggest that scalable, non-clinician-led strategies may have potential for PMS self-management. Future rigorously designed randomized studies with adequate allocation concealment are needed to confirm these findings and to clarify the independent effects of self-monitoring and informational support.

## Supplementary Information

Supplementary Material 1.

## Data Availability

The datasets generated and analyzed during the current study are not publicly available due to privacy considerations and ethical restrictions, but de-identified data may be made available from the corresponding author upon reasonable request and with appropriate institutional approval.

## Abbreviations

PMS: Premenstrual syndrome
PMDD: Premenstrual dysphoric disorder
CBT: Cognitive behavioral therapy
DRSP: Daily Record of Severity of Problems
PSST: Premenstrual Symptoms Screening Tool
CI: Confidence interval
PPS: Per-protocol set
