# Supplementary material for: A digital self-care intervention for psychological distress associated with premenstrual syndrome: a fully online controlled trial using alternating allocation
Source: BMC Womens Health. 2026 May 20;26:350. doi: 10.1186/s12905-026-04539-3 (PMC13361109; doi:10.1186/s12905-026-04539-3)
Supplement: Supplementary file 1 — Supplementary Material 1. [file 12905_2026_4539_MOESM1_ESM.docx]

Supplementary Table 1: Baseline characteristics of participants included in and excluded from the primary per-protocol analysis

|  | | Included in per-protocol analysis N=355 | Excluded from per-protocol analysis N=64 |
| --- | --- | --- | --- |
| Age, mean（SD） | | 31.0 (8.4) | 29.8 (9.6) |
| Medication | Use of psychotropic medication, n (%) | 22 (6.2) | 1 (1.6) |
|  | Use of oral contraceptives, n (%) | 37 (10.4) | 3 (4.7) |
| Operating System | iPhone, n (%) | 267 (75.2) | 58 (90.6) |
|  | Android, n (%) | 88 (24.8) | 6 (9.4) |
| Marital Status Married, n (%) | | 164 (46.2) | 25 (39.1) |
| Employment Status | Full-time, n (%) | 196 (55.2) | 32 (50.0) |
| Education | Student (High school, vocational school, university, graduate school), n (%) | 95 (26.8) | 22 (34.4) |
|  | Middle school graduate, n (%) | 1 (0.3) | 1 (1.6) |
|  | High school graduate, n (%) | 30 (8.5) | 1 (1.6) |
|  | Vocational School/University, Graduate, n (%) | 184 (51.8) | 36 (56.3) |
|  | Postgraduate degree, n (%) | 45 (12.7) | 4 (6.3) |
| PMS classification (PSST) | None - Mild PMS, n (%) | 67 (18.9) | 11 (17.2) |
|  | Moderate PMS, n (%) | 173 (48.7) | 32 (50.0) |
|  | PMDD, n (%) | 115 (32.4) | 21 (32.8) |

*
